# Recovery of the mitochondrial COI barcode region in diverse Hexapoda through tRNA-based primers

**DOI:** 10.1186/1471-2164-11-423

**Published:** 2010-07-09

**Authors:** Doo-Sang Park, Soo-Jung Suh, Hyun-Woo Oh, Paul DN Hebert

**Affiliations:** 1 Biological Resource Center, Korea Research Institute of Bioscience & Biotechnology, Daejeon 305-806, Korea; 2 Industrial Biomaterials Research Center, Korea Research Institute of Bioscience & Biotechnology, Daejeon 305-806, Korea; 3 Youngnam Regional Office, National Plant Quarantine Service, Busan 600-016, Korea; 4 Biodiversity Institute of Ontario, University of Guelph, Guelph, Ontario, N1G 2W1, Canada

## Abstract

**Background:**

DNA barcoding uses a 650 bp segment of the mitochondrial cytochrome *c *oxidase I (COI) gene as the basis for an identification system for members of the animal kingdom and some other groups of eukaryotes. PCR amplification of the barcode region is a key step in the analytical chain, but it sometimes fails because of a lack of homology between the standard primer sets and target DNA.

**Results:**

Two forward PCR primers were developed following analysis of all known arthropod mitochondrial genome arrangements and sequence alignment of the tRNA-W gene which was usually located within 200 bp upstream of the COI gene. These two primers were combined with a standard reverse primer (LepR1) to produce a cocktail which generated a barcode amplicon from 125 of 141 species that included representatives of 121 different families of Hexapoda. High quality sequences were recovered from 79% of the species including groups, such as scale insects, that invariably fail to amplify with standard primers.

**Conclusions:**

A cocktail of two tRNA-W forward primers coupled with a standard reverse primer amplifies COI for most hexapods, allowing characterization of the standard barcode primer binding region in COI 5' as well as the barcode segment. The current results show that primers designed to bind to highly conserved gene regions upstream of COI will aid the amplification of this gene region in species where standard primers fail and provide valuable information to design a primer for problem groups.

## Background

Since 2003, substantial effort has been directed toward the development of a DNA-based identification system for animal life, based upon the analysis of sequence diversity in the 5' region of the mitochondrial gene, cytochrome *c *oxidase 1 [1,2]. Termed DNA barcoding, this approach relies upon the PCR amplification of the target gene region and its subsequent sequence characterization. Performance tests on numerous animal groups have established that the system ordinarily works well - sequence diversity in the 5' region of COI enables discrimination of more than 98% of animal species [3,4]. However, past work has also revealed that standard primer cocktails fail to generate a PCR amplicon in certain taxonomic groups. In some cases, amplification success has been so low that researchers have suggested the need to study slower evolving gene regions that can be recovered more easily [5,6]. Because such movement away from COI represents a serious compromise from the standardization that is fundamental to DNA barcoding, there is much incentive to develop new primer sets that enable recovery of the standard barcode region for 'problem' taxonomic groups.

The Arthropoda represent, by far, the most diverse of animal phyla. Although current primer sets generally perform well, there are some groups where barcode recovery has proven difficult. For example, barcode recoveries in the scale insects (Hemiptera, superfamily Coccoidea) are so low [7] that it has been suggested that an identification system for this group must be developed using another gene. Past efforts to overcome this problem have tried to design primer sets that bind within the COI gene. In the present study, we adopt an alternate approach, one in which the search for primer sites is focused on the tRNA genes that lie upstream of the COI gene in most arthropod mitochondrial genomes. Because these genes have several highly conserved sequence blocks, they have been used as an attractive target for primer design [8,9]. However, there are two major complications that might impede such usage. First, there are a lot of sequence diversities in the conserved blocks of tRNA genes and only short sequences are actually conserved. So, previously developed primers often failed amplify target gene. Another one is the tRNA gene arrangement and its orientation. Individual mitochondrial genes occasionally move from one position in the mitochondrial genome to another. And also their orientation varies from forward to reverse. These difficulties lead to disruption of the design of primers with broad effectiveness. In this study, we strive to identify a tRNA gene whose position and orientation are relatively stable and which represent universality than previously developed primer. Applications of developed primer for COI barcoding the Hexapoda were also discussed.

## Results and Discussion

### Mitochondrial genome analysis, primer design and efficiency test

We began by analyzing gene arrangements for all of the arthropod mitochondrial genomes present in the Mitome database on April 10, 2009 [10]. This analysis re-confirmed the conserved gene arrangement of the three tRNA genes, tRNA-W, tRNA-C and tRNA-Y, ordinarily located upstream of COI in Hexapoda [9]. The position of tRNA-W was particularly consistent as it was found upstream of COI in 120 of 126 hexapod genomes and it was always oriented in a forward direction (Table 1). The six taxa lacking a tRNA-W gene in this position included 3 Phthiraptera, 1 Thysanoptera, 1 Pscoptera and 1 Lepidoptera. The same gene arrangement was also frequently apparent in the mitochondrial genomes of other arthropod subphyla. One or two additional tRNA genes (C, Y) were often present between tRNA-W and the start codon for COI. However, because each of these tRNA genes was only 60-68 bp in length, the tRNA-W gene was invariably located within 200 bp upstream of COI, making it a good target for primer placement.

**Table 1:** Mitochondrial gene arrangements upstream of the COI gene

Phylum	Subphylum	Mitochondrial gene arrangement^a^	Arrangement identified^b^
Arthropoda	Chelicerata	**W,-C,-Y**^c,d,e^; W,-C,-Y,-Y; W,-C; W,-Y; W	33/41
	Crustacea	**W,-C,-Y**; W,-Q,-C (-Y); W,Y; W	29/39
	Hexapoda	**W,-C,-Y**; W,-Y; W,-C; W	120/126
	Myriapoda	**W,-C; W**, W,-Y; W,C,-Y	8/8
Chordata	Vertebrata	**W,-A,-N,-C,-Y**	60/60

^a^, All gene arrangement data are from Mitome http://www.mitome.info/

^b^, Proportion of taxa possessing one of the gene arrangements shown. For example, all 60 vertebrates share one gene order in which four other tRNA genes lie between tRNA-W and COI. By comparison 120 of 126 Hexapoda lineages possess one of four arrangements, none involving more than two intervening tRNA genes

^c^, Dominant gene arrangement is shown in bold

^d^, Reversed gene arrangements are represented as (-)

^e^, Abbreviations for tRNA genes: W - Tryptophan, C- Cysteine, Y- Tyrosine, Q - Glutamine, N- Asparagine and A- Alanine

**Figure 1:**
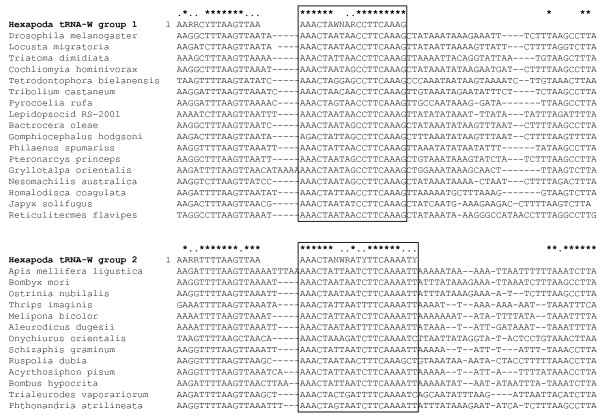
**Alignment of tRNA-W genes from varied Hexapoda**. All 126 Hexapod tRNA-W genes retrieved from GenBank were aligned and manually compared, revealing two groups with high internal sequence homogeneity. Sequence variation within representative species in each group is presented.

**Figure 2:**
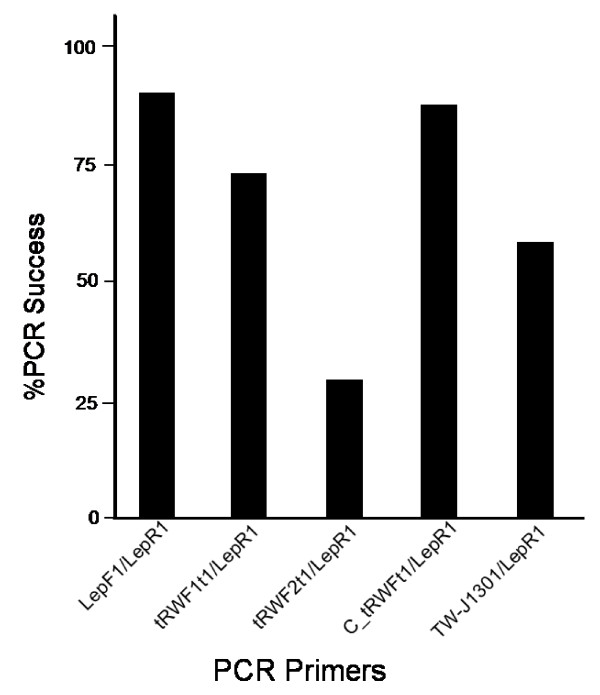
**Amplification success for CO1 using standard primers (LepF1/LepR1) and two new forward primers positioned in the tRNA-W gene in combination with a standard reverse primer (LepR1) both separately and as a cocktail**. TW-J1301 is previously reported tRNA-W primer [9].

Alignment of the tRNA-W sequence from the 120 hexapods revealed two distinct groups of tRNA-W with high internal homogeneity in a central segment of the sequence (Figure 1). The first group (AAACTAWNARCCTTCAAAG) was dominant as it included 92 of the 120 lineages, while the remaining 28 lineages had a slightly divergent sequence (AAACTANWRATYTTCAAAATY). The former group have high sequence homology to previously developed tRNA-W primer, TW-J1301 [9] but the latter showed differences in their central six nucleotides, -NWRATY-. This diversity may explain the previous failure of the tRNA-W primer. Two primers, tRWF1 and tRWF2, designed from these conserved sequences, were performance tested against DNA extracts from 141 species representing 121 different arthropod families in combination with a standard reverse primer (LepR1). As expected for hexapods, there was higher PCR success (Figure 2) for tRWF1 (74%) than for tRWF2 (28%). About 15% of DNA extracts were amplified by both primer sets, while amplification success rose to 87% when the two primers were combined in a 1:1 cocktail. Although this value was slightly lower than the success rate (90%) with the usual arthropod primer set (LepF1, LepR1) the tRWF1&2 cocktail amplified 11 of the 16 samples in our test set that failed with standard primers (Additional file 1). We also examined another universal primer for COI barcoding, LCO1490 and HCO2198 [11], and the PCR recoveries were very similar to that of LepF1/LepR1 primer (data not shown). Because of this complementarity, over 98% success in recovery of a barcode amplicon can be anticipated across all Hexapoda by two PCR reactions, the first using standard primers and the second (for those that failed in the first reaction) employing the primer set developed in this study. The 25 mer primer, TW-J1301, also showed partial complementarity however, it showed only 62% PCR success. This primer has been designed from two conserved regions of tRNA-W gene (7 mer + 18 mer) and the 18 mer-sequence is the same to tRWF1 primer without an additional G on its 3' end. Generally, G or C base located at the 3' end of a PCR primer enhances PCR efficiency and this accounts for higher PCR success for tRWF1 primer than TW-J1301. Because the tRNA primer set enables recovery of the COI sequence from the translation initiation codon, it provides insights into the cause for failed amplifications with the standard primer set, delivering the information needed to design a primer set that will amplify all hexapods.

### DNA barcoding analysis

From the 125 successful PCR amplicons, 111 clean sequences were recovered. The presence of shorter, non-specific amplicons was occasionally noted with the tRNA-W primer set, accounting for some failures in sequence recovery. The total length of the amplicons varied from 730 bp -870 bp. The first 70-200 bp of each read consisted of the sequences for one or more tRNA genes which were originated from different gene arrangement were excised during sequence editing. The remainder of most sequences (107) provided enough coverage for the COI gene to gain formal barcode status (512-711 bp), but four sequences were shorter (279-497 bp). All sequences included 29 bp- 59 bp of sequence information for the far 5' end of the COI gene that was not recovered with the standard primer set, reflecting the sequence region from the presumed initiation codon to the 3' end of the LepF1 primer binding region.

In mitochondria, six kinds of codon, ATN, TTG and GTG have been reported as a canonical translation initiation codon in vertebrate and insect. However, there are several exceptions to this rule, especially in COI gene. Previous reports demonstrated that quadruplets, such as ATAA, TTAA, TTAG, and ATTA could be used as a initiation signal [12-14] although these days, TCG or CGA has more convincing evidence than quadruplets, especially in Diptera [15] and Lepidoptera [16], respectively. In this study, 70 of the 111 sequences began with one of six known arthropod initiation codons (58 ATN, 14 TTG and 1 GTG) and predicted 22 cases, mostly found in Diptera (19/22) with two cases from a Orthoptera (1/1) and a Coleoptera (1/19), using TCG (including a CCG) codon (Additional file 2). Another possible initiator, CGA codon, were found in 17 species distributed in Lepidoptera (9/12), Ephemeroptera (2/5), Diptera (1/22), Mantodea (1/1), Trichoptera (1/5), and a Plecoptera (1/6). These are plausible because a TAG or a TAA stop codon presents at the beginning region of the COI gene and no canonical initiator was found within 30 bp downstream from the stop codon. For two other sequences, the initiation codon was uncertain because sequencing results lacked bidirectional coverage at the 5' end because of presence of short non-specific amplicon which open found at the PCR reaction that used tailed primers.

**Figure 3:**
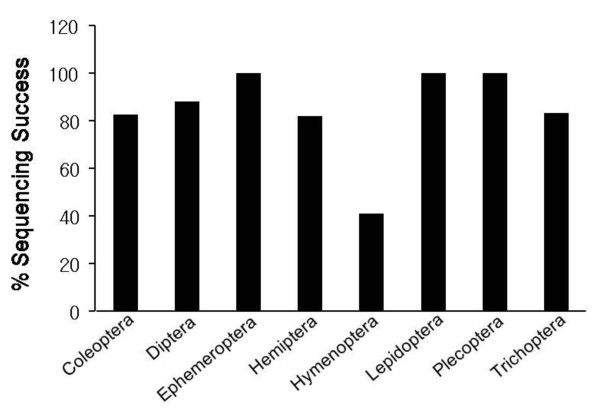
**Success in recovering high quality sequence records from members of 8 insect orders represented by 6 or more different families**. The number of families examined varied among orders: Coleoptera (23), Diptera (25), Ephemeroptera (6), Hemiptera (11), Hymenoptera (17), Lepidoptera (12), Plecoptera (6) and Trichoptera (6).

No indels creating a frame shift or stop codons were present in the COI sequences, suggesting that none derived from a NUMTs. The fact that amplicons were longer than those generated by the standard primer set likely decreased the risk of NUMTs amplification because the latter are usually short [17,18]. Final sequencing success rate varied among the orders (Figure 3). Lepidoptera, Ephemeroptera and Plecoptera showed 100% success (*n *= 12, 6, and 6, respectively), while Hymenoptera showed only 41% sequencing success from 17 samples because PCR generated only 7 clear amplicons. The rest showed weak, non-specific or no amplification (Additional file 1- lane F3 to G7). Diptera, Trichoptera, Hemiptera and Coleoptera (*n *= 25, 6, 11 and 23, respectively) showed 82-88% success. The value for the Hemiptera excluded results for the family Pseucococcidae which only showed 50% success (*n *= 6). The very low PCR and sequencing success in the Hymenoptera, which showed 88% PCR success with the standard primer set, implies high diversity in the location, orientation or sequence of the tRNA-W gene in members of this order, a result already reported for one family of hymenopterans- the Pompilidae [19].

**Figure 4:**
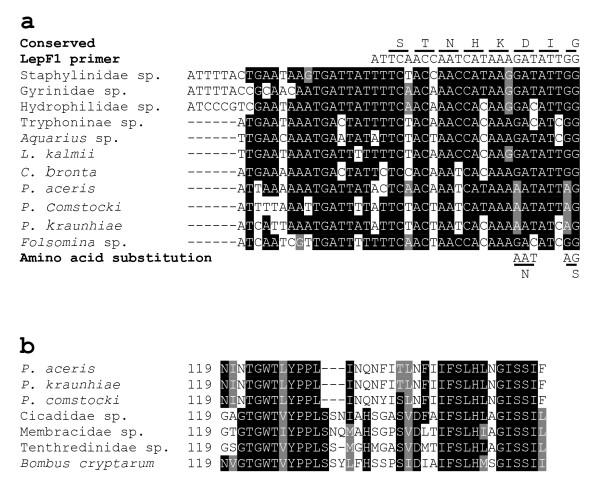
**Sequence characteristics for the 5' region of COI and cases of amino acid deletion**. **a) **Sequence attributes for 11 species that failed to amplify with the standard barcode primers, but that were recovered with the tRNA-W primer cocktail. Sequences are shown from the presumed initiation codon. **b) **Amino acid deletions in three species of Pseudococcidae and a species of Tenthredinidae. The numbers are the amino acid position from the proposed translation initiation codon.

**Figure 5:**
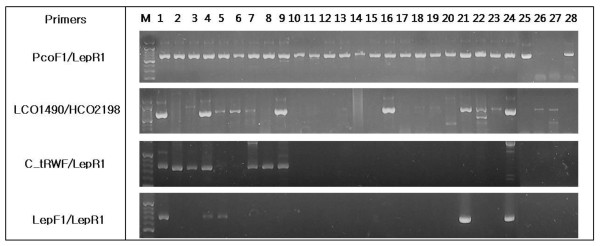
**Gel images of PCR amplicons for 28 scale insect species**. The samples included 9 Pseudococcidae (1-9), 9 Diaspididae (10-18), 6 Coccidae (19-24), 1 Ortheziidae, 1 Eriococcidae, 1 Margarodidae and 1 Conchaspididae.

Sequence analysis of 11 taxa which failed to amplify with standard primers indicated that such failures occurred when more than three nucleotides were substituted and usually involved one or more substitutions within 10 nucleotides of the 3' end (Figure 4a). Most of these substitutions were synonymous, but changes causing amino acid substitutions were detected in the three scale insects, *P. aceris*, *P. kraunhiae *and *P. comstocki*. The G→A transition at the first position of two codons provoked the substitution of Gly→Ser and of Asp→Asn at sites near the 3' end of the primer binding region. These otherwise undetected substitutions explain past failures to recover amplicons for the barcode region in these scale insects using standard primers. We tried to confirm that these substitutions are popular for scale insect species through designing a new forward primer, PcoF1, which reflecting the substitutions and testing its performance for 28 scale insect species. The newly designed primer showed successive amplification for 26 of 28 scale insects samples when combined with the LepR1 primer (Figure 5) while the universal primer sets could only recover 5-6 clear amplicons and the cocktail primer generated amplicons only in Pseucococcidae samples. The 26 successful amplicons includes: 9 Pseudococcidae, 9 Diaspididae, 6 Coccidae, an Ortheziidae and a Conchaspididae species (Additional file 3). The present results confirm the feasibility of creating a comprehensive barcode library for scale insects which previously known as 'difficult barcode group'.

Two additional samples, deriving from species of the wasp family Ichneumonidae (Tryphoninae sp.) and the collembolan family Isotomidae (*Folsomina *sp.), each possessing three T→C substitutions near the 3' end of the primer binding region, represent the only barcode records so far reported for these lineages. The nearest homology group to the species of Tryphoniinae had just 87.6% similarity, while the nearest sequence to the species of *Folsomina *showed just 82.4% congruence.

The alignment of all 111 sequences revealed five cases of amino acid deletion. A block of three amino acids were deleted in three species of Pseudococcidae (Figure 4b). This same deletion occurs in all members of scale insect families tested in this study representing the first case of a three amino acid deletion in COI across all Hexapoda that have been analyzed. Interestingly, there was a single amino acid deletion in a species belonging to the hymenopteran family, Tenthredinidae, at a similar position. A two amino acid deletion was found in a species of Pompilidae (Hymenoptera), but it occurred at a different position in the standard COI barcode region (amino acid position 175 from the initiation codon versus 131/133 amino acids for the scale insects and the tenthrendinid).

As already noted, the new primer cocktail failed to amplify the barcode region in some Hexapoda. Its failure in these cases was likely due to shifts in position of the tRNA-W gene, the existence of intergenic gap between tRNA-W and COI or to sequence variation in the segment of tRNA-W targeted for primer binding. However, this cocktail primer can be a useful supplementary tool for standard COI barcoding method. For example, we could attain 94% of successful barcode from over a thousand of Hemiptera specimens which represents over 200 species with only two PCR amplification steps; the first run, performed with standard primer, obtained only 82% successes. Then the second run conducted with cocktail primer against samples failed at first run (unpublished data). Additionally, this cocktail primer can reduce barcoding failures caused by unexpected amplification of endosymbiont COI where occasionally found in barcoding the Hemiptera.

## Conclusions

The tRNA-W primer cocktail developed in this study successfully amplified the DNA barcode region for most hexapods including many species which failed to generate an amplicon with the standard primer set. Use of this cocktail not only improves the success of barcode recovery, but also provides the information needed to design a primer set for problem groups. The current standard primer set for hexapods are not degenerate, so further study of the upstream sequence of COI promises to aid the development of a new primer cocktail with higher generality.

## Methods

### Mitochondrial genome analysis and primer design

All mitochondrial genome arrangements were assessed using the gene arrangement comparison tool on Mitome, the Mitochondrial Genome Database http://www.mitome.info. tRNA-W gene sequences were retrieved from GenBank and aligned by Clustal W [20]. Some manual modification was done to divide two distinct groups of tRNA-W with high internal homogeneity. Two forward PCR primers: tRWF1 (5'-AAACTAATARCCTTCAAAG-3') and tRWF2 (5'-AAACTAATAATYTTCAAAATTA-3') with M13 tails (5'-TGTAAAACGACGGCCAGT-3') on their 5' end were designed to best represent these two groups and were combined with a standard reverse primer (LepR1) to produce a cocktail (1:1 ratio) which was tested against 141 species representing 121 different families of Hexapoda. A new forward primer for scale insects, PcoF1: 5'- CCTTCAACTAATCATAAAAATATYAG - 3', was designed reflecting sequence diversity in scale insects at the same positions for universal COI barcoding primers.

### DNA analysis

Most of the specimens used in this study derived from collections made by BIO researchers at sites in North America over the past two years. Six scale insect samples were provided from the Central Post-entry Quarantine Station in South Korea. Jointly, these collections included 141 species representing 121 different families of Hexapoda (Additional file 4). All DNA was extracted from either dried or ethanol-fixed leg samples using a standard Glass Fibre extraction protocol [21] except for some small specimens which were processed as whole individuals. PCR thermocycling was done under the following conditions: 2 min at 95°C; 5 cycles of 40 sec at 94°C, 40 sec at 45°C, 70 sec at 72°C; 40 cycles of 40 sec at 94°C, 40 sec at 51°C, 70 sec at 72°C; 5 min at 72°C; held at 4°C.

PCR, PCR check and DNA sequencing were carried out using standard methods[22]. Contigs were assembled using CodonCode aligner Ver2.0.6 (CodonCode Co.) and were subsequently aligned by the same software. The locations of the deletions were identified by manual editing. All sequences have been deposited in GenBank and accession numbers (GU013562 ~ GU013672 and GU936932 ~ GU936957), for the sequences, as well as specimen and collection data, and trace files are available within the DIMC project files in BOLD http://www.barcodinglife.org.

## Authors' contributions

DSP performed laboratory work, led project design and data analysis and co-wrote the manuscript. PDNH aided in design of the project, provided tools/reagents, and co-wrote the manuscript. HWO aided in design of the project and helped in laboratory work. SJS collected and identified the scale insect samples and helped in laboratory work. All authors read and approved the final manuscript.

## Supplementary Material

Additional file 1 **PCR comparisons**. Agarose gels comparing the PCR products generated using both the standard primer set and the tRNA-W primer cocktail. Click here for file

Additional file 2 **Initiation codon of the COI**. Identification of possible initiation codons for sequences amplified by the tRNA-W cocktail was analyzed. Click here for file

Additional file 3 **Scale insect samples used in this study**. Click here for file

Additional file 4 **Information on samples used in this study**. Click here for file
