# Supplementary figures and images for: Recovery of the mitochondrial COI barcode region in diverse Hexapoda through tRNA-based primers
Source: BMC Genomics. 2010 Jul 9;11:423. doi: 10.1186/1471-2164-11-423 (PMC2996951; doi:10.1186/1471-2164-11-423)

## Slide 1
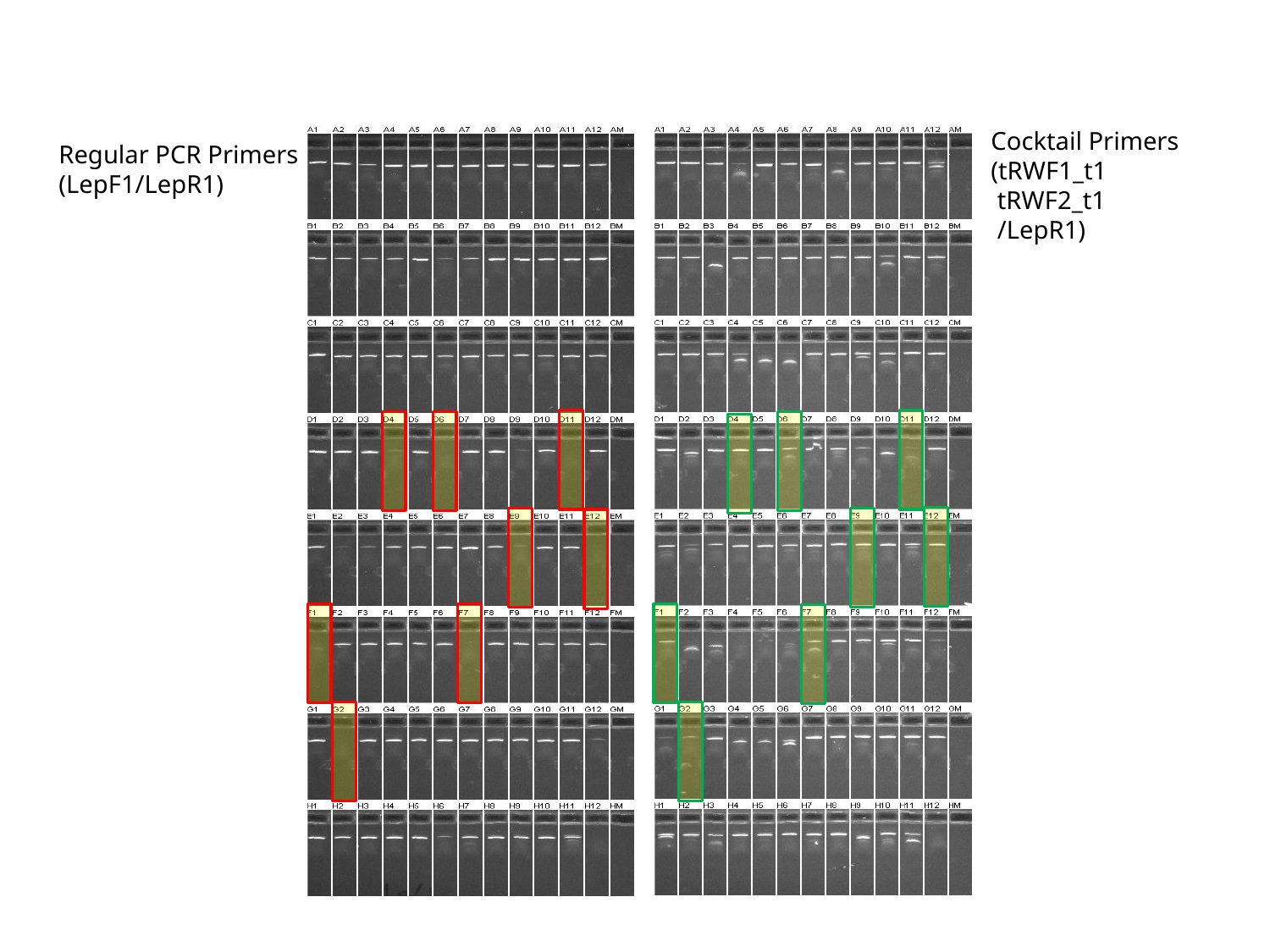

Cocktail Primers
(tRWF1_t1
 tRWF2_t1
 /LepR1)
Regular PCR Primers
(LepF1/LepR1)

## Slide 2
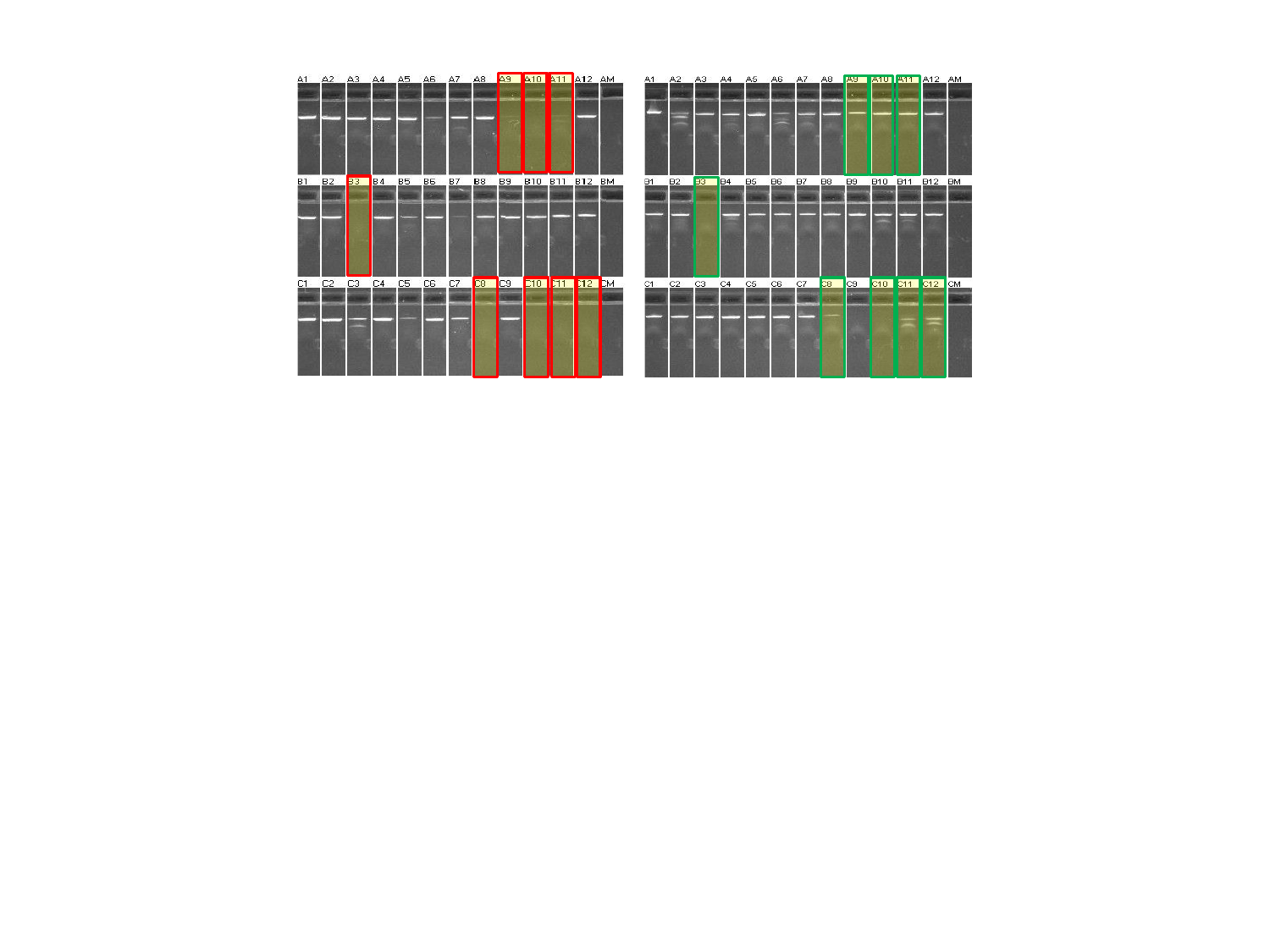

Supplement: Additional file 1 — PCR comparisons. Agarose gels comparing the PCR products generated using both the standard primer set and the tRNA-W primer cocktail. [file 1471-2164-11-423-S1.PPTX]
