# Supplementary material for: Recovery of the mitochondrial COI barcode region in diverse Hexapoda through tRNA-based primers
Source: BMC Genomics. 2010 Jul 9;11:423. doi: 10.1186/1471-2164-11-423 (PMC2996951; doi:10.1186/1471-2164-11-423)
Supplement: Additional file 2 — Initiation codon of the COI. Identification of possible initiation codons for sequences amplified by the tRNA-W cocktail was analyzed. [file 1471-2164-11-423-S2.DOCX]

DIMC001-09 ---------------ATTTAA**TCG**CAACAATGGTTATTCTCTACTAATCATAAAGATATTGG

DIMC002-09 ---------------ATTTAA**TCG**CAACAATGATTGTTCTCAACTAATCATAAGGATATTGG

DIMC003-09 ---------------ATTTAA**TCG**CGACAATGGTTATTTTCAACAAATCATAAAGATATTGG

DIMC005-09 ---------------ATTTAA**TCG**CGACAATGATTATTTTCAACAAATCATAAAGATATTGG

DIMC006-09 ---------------ATTTAA**TCG**CAACAATGATTATTTTCAACTAATCATAAAGATATTGG

DIMC007-09 ---------------ATTTAA**TCG**CAACAATGGCTTTTTTCTACAAACCATAAAGATATTGG

DIMC009-09 ---------------ATTTAA**CCG**CGACAATGATTATTTTCAACAAATCATAAAGATATTGG

DIMC010-09 ---------------ATTTAA**TCG**CAACAATGGCTATTTTCCACTAATCATAAAGATATTGG

DIMC011-09 ---------------TAA**ATA**TTGCAACAATGATTATTTTCTACAAATCATAAAGATATTGG

DIMC012-09 ---------------ATTTAA**TCG**CGACAATGATTATTTTCTACTAATCATAAAGATATTGG

DIMC013-09 ---------------ATTTAA**TCG**CAACAATGGCTATTTTCTACTAATCATAAAGATATTGG Diptera

DIMC014-09 ---------------ATTTAA**TCG**CGAAAATGATTATTTTCTACCAATCATAAAGATATTGG

DIMC016-09 ---------------ATTTAA**TCG**CGACAATGGTTATTTTCTACTAATCATAAAGATATTGG

DIMC017-09 ---------------ATTTAA**TCG**CGACAATGGTTATTCTCTACTAATCATAAAGATATTGG

DIMC018-09 ---------------ATCTAA**TCG**CGACAATGGCTTTTTTCTACAAATCACAAAGATATTGG

DIMC019-09 ---------------ATTTAA**TCG**CGACAATGGTTATTTTCAACTAATCACAAAGATATTGG

DIMC020-09 ---------------TTTAAAACG**CGA**CAATGATTATTTTCAACAAATCATAAGGATATTGG

DIMC021-09 ---------------ATTTAA**TCG**CGACAATGGCTGTTTTCAACAAATCATAAAGATATTGG

DIMC022-09 ---------------ATTTAA**TCG**CAACAATGGTTATTTTCAACAAATCATAAAGATATTGG

DIMC023-09 ---------------ATTTAA**TCG**CGACAGTGATTATTTTCAACAAACCATAAAGATATTGG

DIMC024-09 ---------------**ATT**CAATCGCAACAATGGTTATTCTCTACTAATCATAAAGATATTGG

DIMC025-09 ---------------ATCTAA**TCG**CGACAGTGATTATTTTCAACAAATCATAAAGATATTGG

DIMC026-09 ---------------------**ATG**AAACGATGATTATTCTCAACAAATCATAAAGATATTGG Phasmatodea

DIMC027-09 ---------------ACTTTA**TCG**CAACGATGATTATTTTCTACTAATCATAAAGACATTGG Orthoptera

DIMC031-09 ---------------CTTACTCTGCAA**CGA**TGATTATTCTCAACTAATCACAAAGACATTGG Mantodea

DIMC032-09 ------------**ATT**TTACCATCGCGACAATGATTATTTTCAACTAACCATAAGGATATTGG

Neuroptera

DIMC034-09 ---**ATT**CAGCCATTTTACCTTACGCAAAAATGATTATTCTCAACTAACCACAAGGATATTGG

DIMC035-09 ---------------------**TTG**AAACGATGATTATTTTCAACAAATCATAAAGACATTGG Blattaria

DIMC036-09 ---------------**ATT**TTACTGAAAAAATGACTCTACTCAACAAACCATAAAGATATTGG Raphidioptera

DIMC037-09 ---------------------**GTG**CGAAAATGGCTTTTTTCTACAAATCATAAAGATATTGG Mecoptera

DIMC039-09 ---------------------**TTG**AAGCGATGATTATTCTCAACAAATCACAAAGACATTGG Dermaptera

DIMC040-09 ---------------**ATC**CTATCGAACAAATGACTATTTTCGACAAACCACAAGGATATTGG

DIMC041-09 ------------**ATT**TCACCCTCGAATAAATGATTATATTCAACAAACCACAAAGATATTGG

DIMC042-09 ---------------**ATT**TTACTGAATAAGTGATTATTTTCTACCAACCATAAGGATATTGG

DIMC043-09 ---------------**ATT**TTACCGCGACAATGATTATTTTCAACAAACCATAAGGATATTGG

DIMC044-09 ---------------------**ATG**AACAAATGACTCTTCTCCACCAATCACAAAGATATTGG

DIMC045-09 ---------------ACTTTA**TCG**AATAAATGATTATTTTCTTCTAATCATAAAGATATTGG

DIMC048-09 ---------------**ATT**CTACTAAACAAATGATTTTTCTCTACAAACCATAAAAATATTGG

DIMC049-09 ---------------**ATT**TTACCGAATAAATGATTCTATTCTACAAATCATAAAGATATTGG

DIMC051-09 ---------------**ATT**TCACTTAATAAATGATTATTCTCTACAAATCATAAAGATATTGG

DIMC052-09 ---------------**ATT**TTACCGAATAAATGACTTTTTTCTACAAACCATAAAAATATTGG

DIMC053-09 ---------------**ATT**CTACTGAACAAATGACTATTTTCGACTAACCATAAGGATATTGG Coleoptera

DIMC054-09 ---------------------**ATG**AATAAATGATTATTTTCAACAAATCACAAAGATATTGG

DIMC055-09 ------------------**ATA**TCAATAAAATGACTTTTCTCTACAAATCATAAAGACATTGG

DIMC056-09 ---------------------**ATG**AATAAATGATTATTTTCCACAAATCATAAGGATATTGG

DIMC057-09 ---------------**ATT**TTATCGAATAAATGATTTTTATCAACTAACCATAAAGAAATTGG

DIMC058-09 ---------------------**TTG**AATAAGTGATTATTTTCAACAAACCACAAAGACATTGG

DIMC059-09 ---------------**ATC**TCATCGAATAAATGATTATTTTCAACAAACCACAAAGATATCGG

DIMC060-09 ---------------**ATT**TTACCGCAACAATGATTATTTTCAACAAACCATAAGGATATTGG

DIMC061-09 ---------------**ATC**CCGTCGAATAAATGATTATTTTCAACAAACCACAAGGACATTGG

DIMC068-09 ---------------------**TTG**ACAAAATGATTATATTCAACTAATCATAAAGATATTGG

DIMC069-09 ---------------------**ATG**AATAAATGACTATTTTCTACAAACCACAAAGACATCGG

DIMC070-09 ---------------------**ATG**AACAAATGATTATTTTCTACTAATCATAAAAATATTGG

DIMC071-09 ---------------------**TTG**AATAAATGATTATTTTCTACTAATCATAAAAATATTGG Hymenoptera

DIMC072-09 ---------------------**ATG**AATAAATGATTATATTCCACAAATCATAAAGATATCGG

DIMC075-09 ---------------------**ATA**AAAAAATGATTAATATCAACTAACCATAAAAATATTGG

DIMC079-09 ---------------------**ATG**AAAAAATGATTGATATCAACTAATCATAAAGATATTGG

DIMC080-09 ---------------ATTAATTAG**CGA**AAATGACTTTATTCTACTAATCATAAAGATATTGG

DIMC081-09 ---------------TATTATTAG**CGA**AAATGATTATTCTCTACAAATCATAAAGATATTGG

DIMC082-09 ---------------TTTTATTAG**CGA**AAATGACTTTATTCTACAAATCATAAAGATATTGG

DIMC083-09 ---------------TATTTAAAG**CGA**AAATGACTTTATTCAACTAACCATAAAGATATTGG

DIMC084-09 ---------------ATTAAATAG**CGA**AAATGACTTTTTTCAACAAATCATAAAGATATTGG

DIMC085-09 ---------------**ATT**TTATTACGAAAATGACTTTACTCTACAAATCATAAAGATATTGG Lepidoptera

DIMC086-09 ---------------TTATTTAAG**CGA**AAATGACTTTATTCAACAAATCATAAAGATATTGG

DIMC087-09 ---------------**ATT**TTTACGCGAAAATGACTTTACTCTACAAATCATAAAGATATTGG

DIMC088-09 ---------------TTTATTTAG**CGA**AAATGACTTTATTCAACAAATCATAAAGATATTGG

DIMC089-09 ---------------TTTTAAAAG**CGA**AAATGACTTTTTTCAACAAATCATAAAGATATTGG

DIMC090-09 ---------------TATTTCAAG**CGA**AAATGATTTTTTTCTACTAATCATAAAGATATCGG

DIMC091-09 ---------------------**TTG**CGAAAATGACTTTTTTCTACAAATCATAAGGATATTGG

DIMC092-09 ---------------------**ATG**CGACAATGACTTTTTTCAACTAATCACAAAGACATTGG

DIMC094-09 ---------------------**TTG**CGACAATGACTTTTTTCTACAAACCATAAGGATATTGG Odonata

DIMC095-09 ---------------------**TTG**CAACGATGGCTATTTTCTACTAATCATAAGGATATTGG

DIMC096-09 ---------------**ATT**CTATCGCGAAAATGATTATTTTCTACAAATCACAAAGATATTGG

DIMC097-09 ---------------**ATT**CTACCGCAAAAATGATTATTTTCAACAAATCATAAAGATATTGG Megaloptera

DIMC098-09 ---------------**ATT**TTACCGCGAAAATGGTTATTTTCTACAAATCATAAAGATATTGG

DIMC100-09 ---------------------**TTG**AATAAATGATTATATTCTACAAATCATAAAGACATTGG

DIMC102-09 ---------------------**TTG**AATAAATGATTATTTTCAACAAATCATAAAAACATCGG

DIMC103-09 ---------------------**ATG**AATAAATGACTATTTTCAACTAACCACAAAGATATTGG

DIMC104-09 ---------------------**TTG**AATAAATGATTATACTCAACCAATCATAAAGATATTGG

DIMC105-09 ---------------------**TTG**AACAAATGAATATATTCTACTAACCACAAAGATATCGG Hemiptera

DIMC106-09 ---------------------**TTG**AATAAATGATTTTTTTCTACAAACCACAAGGATATTGG

DIMC107-09 ---------------------**ATG**TATAAATGAGTATTCTCAACCAACCACAAGGATATCGG

DIMC108-09 ---------------------**ATG**AATAAATGACTATTCTCAACAAACCACAAAGACATTGG

DIMC109-09 ---------------------**ATG**AAAAAATGGCTGTTTTCCACTAATCATAAGGACATTGG

DIMC111-09 ---------------------**ATT**AAAAAATGAATATTTTCAACAAATCATAAAATTATTGG

DIMC112-09 ---------------------**ATT**AAACAATGATTATTTTCTACAAACCACAAAGATATTGG

DIMC113-09 ---------------**ATT**TTACTGCGACAATGAATATTTTCAACAAATCATAAAGATATTGG Trichoptera

DIMC114-09 ---------------------**ATG**AAAAAATGACTATTCTCCACAAATCACAAAGATATTGG

DIMC115-09 ---------------AATTTACTG**CGA**CAATGATTATTTTCTACAAATCATAAAGATATCGG

DIMC116-09 ---------------**ATT**CTACCGCGACAATGATTATTTTCAACAAATCATAAGGATATTGG

DIMC117-09 ---------------**ATT**CTACCGCGACAATGATTATTTTCAACAAATCATAAGGACATTGG

DIMC118-09 ---------------**ATT**TTACCGCGACAATGATTATTTTCAACAAACCATAAGGACATTGG

DIMC119-09 ---------------TTCTACCCG**CGA**CAATGATTATTTTCAACAAACCATAAGGACATTGG Plecoptera

DIMC120-09 ---------------------**ATG**CGACAATGGTTGTTCTCAACCAATCACAAAGACATTGG

DIMC121-09 ---------------------**ATG**CGACAATGGCTTTTCTCAACAAATCACAAAGATATTGG

DIMC122-09 ---------------ACTTTACCG**CGA**CAATGATTATATTCAACAAACCACAAGGACATTGG

DIMC123-09 ---------------------**ATG**CGACAATGATTATATTCAACAAACCATAAGGACATTGG

DIMC124-09 ---------------**ATA**TTATCGCGACAATGACTATTTTCAACAAACCATAAAGATATTGG Ephemeroptera

DIMC125-09 ---------------------CCG**CGA**CAATGATTATTTTCAACAAACCATAAGGATATTGG

DIMC126-09 ---------------------**ATG**CGACAATGATTATTCTCAACAAACCATAAGGATATTGG

DIMC131-09 ---------------------**ATT**TTAAATTGATTTTATTCTACTAATCATAAAAATATTAG

DIMC132-09 ---------------------**ATC**ATTAAATGATTATATTCTACTAATCACAAAAATATCAG Hemiptera

DIMC133-09 ---------------------**ATT**AAAAAATGATTATACTCAACAAACCATAAAAATATTAG

DIMC138-09 ---------------------**ATG**AAACGTTGACTTTTTTCAACCAACCATAAAGATATTGG

DIMC140-09 ---------------------**ATG**AAACGTTGAGTTTTTTCAACCAACCATAAAGATATTGG Entomobryomorpha

DIMC143-09 ---------------------**ATC**AATCGTTGATTTTTTTCAACTAACCACAAAGACATCGG

DIMC139-09 ---------------------------------**ATC**TTCTCGACTAACCATAAAGATATTGG

DIMC141-09 ---------------------------------**ATC**TTCTCGACTAACCATAAAGATATTGG Poduromorpha

DIMC142-09 ---------------------------------**ATC**TTCTCGACTAACCATAAAGATATTGG
