# Supplementary material for: Recovery of the mitochondrial COI barcode region in diverse Hexapoda through tRNA-based primers
Source: BMC Genomics. 2010 Jul 9;11:423. doi: 10.1186/1471-2164-11-423 (PMC2996951; doi:10.1186/1471-2164-11-423)
Supplement: Additional file 3 — Scale insect samples used in this study. [file 1471-2164-11-423-S3.DOCX]

**Table .** Collection details of scale insects

| **Family** | **Species** | **Label** | **Sample ID** | **Country** | **Collection date** | **Host** | **GenBank Accession** |
| --- | --- | --- | --- | --- | --- | --- | --- |
| Pseudococcidae | *Pseudococcus comstocki* (Kuwana) | 1 | 20070229 | South Korea | 26 Oct. 2007 | *Pyrus* | GU936932 |
|  | *Crisicoccus matsumotoi* (Siraiwa) | 2 | P1018-5 | Japan | 2007 (reared) | *Diospyros* | GU936933 |
|  | *Planococcus kraunhiae* (Kuwana) | 3 | P1016-2 | Japan | 6 Apr. 1997 (reared) | *Diospyros* | GU936934 |
|  | *Phenacoccus aceris* (Signoret) | 4 | K20080058 | South Korea | 18 Apr. 2008 | *Zelkova* | GU936935 |
|  | *Dysmicoccus brevipes* (Cockerell) | 5 | P1192-1 | Thailand | 25 Mar. 2009 | *Pandanus* | GU936936 |
|  | *Phenacoccus solani* (Ferris) | 6 | P1110-1 | Japan | 06 Nov. 2008 | *Echeveria* | GU936937 |
|  | *Planococcus citri* (Risso) | 7 | K20080001 | South Korea | 28 Feb. 2008 | *Opuntia* | GU936938 |
|  | *Heliococcus kurilensis* Danzig | 8 | 20090073 | ;South Korea | 14 May 2009 | *Rubus* | GU936939 |
|  | *Coccura comari* (Kunow) | 9 | 20090099 | South Korea | 20 May 2009 | *Rubus* | GU936940 |
| Diaspididae | *Aonidiella aurantii* (Maskell) | 10 | D549-2 | United States | 13 Sep. 2008 | *Citrus* | GU936941 |
|  | *Aspidiotus destructor* Signoret | 11 | D581 | Malaysia | 29 Mar. 2009 | *Cocos* | GU936941 |
|  | *Aulacaspis spinosa* (Maskell) | 12 | K20050047 | South Korea | 19 Oct. 2005 | *Smilax* | GU936943 |
|  | *Chrysomphalus aonidum* (Linnaeus) | 13 | D538-1 | Indonesia | 29 Apr. 2008 | *Dracaena* | GU936944 |
|  | *Kuwanaspis hikosani* (Kuwana) | 14 | K20090032-1 | South Korea | 02 Apr. 2009 | *Pseudosasa* | GU936945 |
|  | *Lepidosaphes pini* (Maskell) | 15 | K20090004-1 | South Korea | 01 Apr. 2009 | *Pinus* | GU936946 |
|  | *Lopholeucaspis japonica* (Cockerell) | 16 | K20090025 | South Korea | 02 Apr. 2009 | *Euonymus* | GU936947 |
|  | *Pinnaspis strachani* (Cooley) | 17 | D566 | Costa Rica | 14 Jan. 2009 | *Dracaena* | GU936948 |
|  | *Pseudaulacaspis cockerelli* (Cooley) | 18 | K20090035-1 | South Korea | 02 Apr. 2009 | *Forsythia* | GU936949 |
| Coccidae | *Coccus hesperidum* Linnaeus | 19 | C31 | China | 24 Oct. 2008 | *Areca* | GU936950 |
|  | *Kilifia acuminata* (Signoret) | 20 | C36 | Egypt | 22 Jul. 2006 | *Ficus* | GU936951 |
|  | *Pulvinariella mesembryanthemi* (Vallot) | 21 | C40 | Australia | 26 Feb. 2009 | *Mesembryanthemum* | GU936952 |
|  | *Coccus viridis* (Green) | 22 | C43 | Thailand | 18 Mar. 2009 | *Citrus* | GU936953 |
|  | *Saissetia oleae* (Olivier) | 23 | C53 | Japan | 18 Jan..2010 | *Ficus* | GU936954 |
|  | *Ceroplastes ceriferus* (Fabricius) | 24 | 20100005 | South Korea | 05 Feb. 2010 | *Camellia* | GU936955 |
| Ortheziidae | *Orthezia urticae* (Linnaeus) | 25 | 20090117 | South Korea | 29 May 2009 | *Artemisia* | GU936956 |
| Eriococcidae | *Asiacornococcus kaki* (Kuwana) | 26 | 20060315 | South Korea | 15 Sep. 2006 | *Diospyros* |  |
| Margarodidae | *Icerya purchasi* Maskell | 27 | 20060292 | South Korea | 22 Aug 206 | *Psidium* |  |
| Conchaspididae | *Conchaspis cordiae* Mamet | 28 | M10 | South Korea | 26 May 2005 | *Swietenia* | GU936957 |
