# Supplementary material for: Recovery of the mitochondrial COI barcode region in diverse Hexapoda through tRNA-based primers
Source: BMC Genomics. 2010 Jul 9;11:423. doi: 10.1186/1471-2164-11-423 (PMC2996951; doi:10.1186/1471-2164-11-423)
Supplement: Additional file 4 — Information on samples used in this study. [file 1471-2164-11-423-S4.DOC]

|  |  |  |  | BOLD | GenBank |
| --- | --- | --- | --- | --- | --- |
| Family | Identification | Collection locality | Voucher ID | Process ID | Accession number |
| Sarcophagidae | | Canada, Manitoba | 08BBDIP-0018 | DIMC001-09 | GU013656 |
| Syrphidae |  | Canada, Manitoba | 08BBDIP-0044 | DIMC002-09 | GU013663 |
| Bombyliidae |  | Canada, Saskatchewan | 08BBDIP-0062 | DIMC003-09 | GU013573 |
| Stratiomyidae | Stratiomys | Canada, Manitoba | 08BBDIP-0096 | DIMC004-09 |  |
| Tabanidae | Hybomitra nuda | Canada, Manitoba | 08BBDIP-0011 | DIMC005-09 | GU013605 |
| Empididae |  | Canada, Manitoba | 08BBDIP-0299 | DIMC006-09 | GU013595 |
| Sciomyzidae |  | Canada, Alberta | 08BBDIP-1097 | DIMC007-09 | GU013658 |
| Asilidae |  | Canada, Ontario | 08BBDIP-0355 | DIMC008-09 |  |
| Rhagionidae | Rhagio mystaceus | Canada, Ontario | 08BBDIP-0229 | DIMC009-09 | GU013655 |
| Tipulidae | Nephrotoma | Canada, Saskatchewan | 08BBDIP-0178 | DIMC010-09 | GU013633 |
| Chironomidae | Chironomus entis | Canada, Manitoba | 08BBDIP-0204 | DIMC011-09 | GU013581 |
| Tachinidae |  | Canada, Manitoba | 08BBDIP-0420 | DIMC012-09 | GU013664 |
| Muscidae |  | Canada, Manitoba | 08BBDIP-0014 | DIMC013-09 | GU013629 |
| Therevidae |  | Canada, Saskatchewan | 08BBDIP-0459 | DIMC014-09 | GU013668 |
| Sciomyzidae |  | Canada, Saskatchewan | 08BBDIP-0132 | DIMC015-09 |  |
| Calliphoridae | Phormia regina | Canada, Manitoba | 08BBDIP-0310 | DIMC016-09 | GU013648 |
| Dolichopodidae | | Canada, Manitoba | 08BBDIP-0311 | DIMC017-09 | GU013594 |
| Ulidiidae | Tritoxa | Canada, Saskatchewan | 08BBDIP-0174 | DIMC018-09 | GU013670 |
| Sepsidae | Sepsis neocynipsea | Canada, Alberta | 08BBDIP-1456 | DIMC019-09 | GU013659 |
| Conopidae |  | Canada, Alberta | 08BBDIP-1290 | DIMC020-09 | GU013590 |
| Pyrgotidae | Sphecomyiella valida | Canada, Ontario | 08BBDIP-1772 | DIMC021-09 | GU013662 |
| Tephritidae | Urophora cardui | Canada, Ontario | 08FAYTT-0071 | DIMC022-09 | GU013671 |
| Culicidae | Coquillettidia perturbans | Canada, Ontario | 08MZPP-088 | DIMC023-09 | GU013591 |
| Mydidae | Mydas clavatus | Canada, Ontario | 08MZPP-084 | DIMC024-09 | GU013630 |
| Micropezidae |  | Canada, Ontario | 08MZPP-171 | DIMC025-09 | GU013628 |
| Heteronemiidae | Diapheromera femorata | Canada, Ontario | 08MZPP-031 | DIMC026-09 | GU013592 |
| Gryllidae |  | Canada, Saskatchewan | 08BBORTH-0004 | DIMC027-09 | GU013600 |
| Acrididae |  | Canada, Saskatchewan | 08BBORTH-0061 | DIMC028-09 |  |
| Tettigoniidae |  | Canada, Manitoba | 08BBORTH-0386 | DIMC029-09 |  |
| Amorphoscelidae |  | Australia, New South Wales | 08-NSWHH-1410 | DIMC030-09 |  |
| Mantidae |  | Australia, New South Wales | 08-NSWHH-1405 | DIMC031-09 | GU013622 |
| Hemerobiidae |  | Canada, Ontario | 08BBNEU-005 | DIMC032-09 | GU013603 |
| Chrysopidae |  | Canada, Ontario | 08BBNEU-008 | DIMC033-09 | GU013582 |
| Myrmeleontidae | | Canada, Saskatchewan | 08BBNEU-022 | DIMC034-09 | GU013631 |
| Blattellidae | Parcoblatta pennsylvanica | Canada, Ontario | 08BBBLA-001 | DIMC035-09 | GU013646 |
| Raphidiidae | Agulla | Canada, Alberta | 08BBRAPH-001 | DIMC036-09 | GU013564 |
| Panorpidae | Panorpa | Canada, Ontario | 08BBMEC-004 | DIMC037-09 | GU013640 |
| Pygidicranidae |  | Australia, Queensland | 08INLI-071 | DIMC038-09 |  |
| Kalotermitidae |  | Australia, New South Wales | 08-NSWHH-1411 | DIMC039-09 | GU013610 |
| Scarabaeidae |  | Canada, Ontario | 08SOCOL-0019 | DIMC040-09 | GU013589 |
| Silphidae | Nicrophorus | Canada, Ontario | 08SOCOL-0023 | DIMC041-09 | GU013634 |
| Staphylinidae |  | Canada, Ontario | 08SOCOL-0029 | DIMC042-09 | GU013588 |
| Carabidae |  | Canada, Ontario | 08SOCOL-0052 | DIMC043-09 | GU013578 |
| Scarabaeidae |  | Canada, Ontario | 08SOCOL-0087 | DIMC044-09 | GU013657 |
| Coccinellidae | Coccinella septempunctata | Canada, Ontario | 08SOCOL-0102 | DIMC045-09 | GU013586 |
| Chrysomelidae | | Canada, Ontario | 08SOCOL-0103 | DIMC046-09 |  |
| Elateridae |  | Canada, Ontario | 08SOCOL-0095 | DIMC047-09 |  |
| Lampyridae |  | Canada, Ontario | 08SOCOL-0096 | DIMC048-09 | GU013614 |
| Brentidae |  | Canada, Ontario | 08SOCOL-0148 | DIMC049-09 | GU013575 |
| Curculionidae | Polydrusus | Canada, Ontario | 08SOCOL-0192 | DIMC050-09 |  |
| Cantharidae |  | Canada, Ontario | 08SOCOL-0233 | DIMC051-09 | GU013577 |
| Cerambycidae | Anthophylax attenuatus | Canada, Ontario | 08BBCOL-0023 | DIMC052-09 | GU013568 |
| Meloidae |  | Canada, Saskatchewan | 08BBCOL-0100 | DIMC053-09 | GU013626 |
| Cleridae |  | Canada, Saskatchewan | 08BBCOL-0150 | DIMC054-09 | GU013585 |
| Lycidae | Dictyoptera | Canada, Ontario | 08BBCOL-0193 | DIMC055-09 | GU013593 |
| Buprestidae | Agrilus planipennis | Canada, Ontario | 08MZPP-129 | DIMC056-09 | GU013563 |
| Erotylidae | Megalodacne fasciata | Canada, Ontario | 08MZPP-071 | DIMC057-09 | GU013623 |
| Tenebrionidae | Bolitotherus cornutus | Canada, Ontario | 08MZPP-006 | DIMC058-09 | GU013570 |
| Hydrophilidae |  | Canada, Ontario | 08MZPP-168 | DIMC059-09 | GU013587 |
| Gyrinidae |  | Canada, Ontario | 08OEC-181 | DIMC060-09 | GU013601 |
| Hydrophilidae |  | Canada, Ontario | 08SOCOL-0111 | DIMC061-09 | GU013606 |
| Phengodidae |  | Canada, Ontario | 08SOCOL-0356 | DIMC062-09 |  |
| Halictidae |  | Canada, Saskatchewan | 08BBHYM-0013 | DIMC063-09 |  |
| Sphecidae |  | Canada, Saskatchewan | 08BBHYM-0026 | DIMC064-09 |  |
| Crabronidae |  | Canada, Saskatchewan | 08BBHYM-0042 | DIMC065-09 |  |
| Chrysididae |  | Canada, Saskatchewan | 08BBHYM-0048 | DIMC066-09 |  |
| Mutillidae |  | Canada, Saskatchewan | 08BBHYM-0074 | DIMC067-09 |  |
| Vespidae |  | Canada, Ontario | 08BBHYM-0083 | DIMC068-09 | GU013672 |
| Ichneumonidae | Tryphoninae | Canada, Manitoba | 08BBHYM-0636 | DIMC069-09 | GU013608 |
| Tenthredinidae | Tenthredo sp. | Canada, Alberta | 08BBHYM-0294 | DIMC070-09 | GU013666 |
| Tenthredinidae |  | Canada, Ontario | 08BBHYM-0082 | DIMC071-09 | GU013665 |
| Formicidae | Formica obscuripes | Canada, Saskatchewan | 08BBHYM-0682 | DIMC072-09 | GU013598 |
| Megachilidae | Osmia subaustralis | Canada, Ontario | 08BBHYM-0694 | DIMC073-09 |  |
| Eurytomidae | Eurytoma | Canada, Alberta | 08BBHYM-1248 | DIMC074-09 |  |
| Apidae | Bombus cryptarum | Canada, Alberta | 08BBHYM-0305 | DIMC075-09 | GU013572 |
| Siricidae | Urocerus flavicomis | Canada, Alberta | 08BBHYM-0384 | DIMC076-09 |  |
| Perilampidae | Perilampus | Canada, Saskatchewan | 08BBHYM-0058 | DIMC077-09 |  |
| Pompilidae |  | Canada, Manitoba | 08BBHYM-0047 | DIMC078-09 |  |
| Pompilidae |  | Canada, Saskatchewan | 08BBHYM-0143 | DIMC079-09 | GU013607 |
| Saturniidae | Antheraea polyphemus | Canada, Ontario | 08BBLEP-00225 | DIMC080-09 | GU013567 |
| Yponomeutidae | Atteva punctella | Canada, Ontario | 08MZPP-114 | DIMC081-09 | GU013569 |
| Papilionidae | Papilio canadensis | Canada, Ontario | 08BBLEP-00006 | DIMC082-09 | GU013642 |
| Pieridae | Pieris napi | Canada, Saskatchewan | 08BBLEP-00010 | DIMC083-09 | GU013649 |
| Lycaenidae | Celastrina ladon | Canada, Ontario | 08BBLEP-00177 | DIMC084-09 | GU013579 |
| Sphingidae | Hemaris diffinis | Canada, Ontario | 08BBLEP-00002 | DIMC085-09 | GU013602 |
| Geometridae | Tetracis cachexiata | Canada, Ontario | 08BBLEP-00140 | DIMC086-09 | GU013667 |
| Sphingidae | Paonias excaecata | Canada, Ontario | 08BBLEP-00112 | DIMC087-09 | GU013641 |
| Arctiidae | Platarctia parthenos | Canada, Ontario | 08BBLEP-00175 | DIMC088-09 | GU013651 |
| Pyralidae | Pyla fusca | Canada, Ontario | 08BBLEP-00178 | DIMC089-09 | GU013654 |
| Pterophoridae | Paraplatyptilia | Canada, Manitoba | 08BBLEP-00871 | DIMC090-09 | GU013645 |
| Nymphalidae | Limenitis arthemis | Canada, Ontario | PPBP-2446 | DIMC091-09 | GU013618 |
| Lestidae | Lestes unguiculatus | Canada, Alberta | 08BBODO-011 | DIMC092-09 | GU013617 |
| Coenagrionidae | Ischnura | Canada, Alberta | 08BBODO-012 | DIMC093-09 |  |
| Aeshnidae | Aeshna constricta | Canada, Alberta | 08BBODO-016 | DIMC094-09 | GU013562 |
| Corduliidae | Somatochlora franklini | Canada, Alberta | 08BBODO-018 | DIMC095-09 | GU013661 |
| Corydalidae | Nigronia serricornis | Canada, Ontario | 08BBMEG-003 | DIMC096-09 | GU013635 |
| Corydalidae | Chauliodes | Canada, Ontario | 08BBMEG-002 | DIMC097-09 | GU013625 |
| Sialidae |  | Canada, New Brunswick | 08NBINS-0029 | DIMC098-09 | GU013624 |
| Corixidae | Sigara decoratella | Canada, Manitoba | 08BBHEM-138 | DIMC099-09 |  |
| Notonectidae | Notonecta borealis | Canada, Manitoba | 08BBHEM-163 | DIMC100-09 | GU013636 |
| Miridae | Leptopterna dolabrata | Canada, Alberta | 08BBHEM-047 | DIMC101-09 |  |
| Nabidae | Nabicula subcoleoptrata | Canada, Saskatchewan | 08BBHEM-226 | DIMC102-09 | GU013632 |
| Cicadellidae |  | Canada, Manitoba | 08BBHEM-124 | DIMC103-09 | GU013583 |
| Pentatomidae |  | Canada, Saskatchewan | 08BBHEM-222 | DIMC104-09 | GU013669 |
| Gerridae | Aquarius | Canada, Ontario | 08BBHEM-184 | DIMC105-09 | GU013599 |
| Lygaeidae | Lygaeus kalmii | Canada, Saskatchewan | 08BBHEM-240 | DIMC106-09 | GU013621 |
| Dictyopharidae | Scolops | Canada, Manitoba | 08BBHEM-123 | DIMC107-09 | GU013627 |
| Membracidae |  | Canada, Alberta | 08BBHEM-106 | DIMC108-09 | GU013584 |
| Cicadidae |  | Canada, Saskatchewan | 08BBHEM-219 | DIMC109-09 | GU013620 |
| Hydropsychidae | Cheumatopsyche harwoodi | Canada, Ontario | 07ONCAD-0103 | DIMC110-09 |  |
| Polycentropodidae | Polycentropus cinereus | Canada, Manitoba | 08MBCAD-0246 | DIMC111-09 | GU013652 |
| Lepidostomatidae | Lepidostoma togatum | Canada, Manitoba | 08MBCAD-0404 | DIMC112-09 | GU013615 |
| Brachycentridae | Brachycentrus americanus | Canada, Alberta | 08ABCAD-0211 | DIMC113-09 | GU013574 |
| Hydropsychidae | Ceratopsyche bronta | Canada, Manitoba | 08MBCAD-0236 | DIMC114-09 | GU013580 |
| Limnephilidae | Limnephilus externus | Canada, Alberta | 08ABCAD-0184 | DIMC115-09 | GU013619 |
| Capniidae | Allocapnia granulata | United States, New York | 08BKEPT-250 | DIMC116-09 | GU013565 |
| Taeniopterygidae | Bolotoperla rossi | United States, New York | 08BKEPT-275 | DIMC117-09 | GU013571 |
| Leuctridae | Paraleuctra sara | United States, New York | 08BKEPT-294 | DIMC118-09 | GU013643 |
| Nemouridae | Paranemoura perfecta | United States, New York | 08BKEPT-300 | DIMC119-09 | GU013644 |
| Chloroperlidae | Alloperla severa | United States, Montana | 08BKEPT-306 | DIMC120-09 | GU013566 |
| Perlodidae | Isoperla fulva | United States, Montana | 08BKEPT-316 | DIMC121-09 | GU013609 |
| Ephemeridae | Ephemera simulans | Canada, Ontario | 08ONMAY-0023 | DIMC122-09 | GU013596 |
| Ephemeridae | Hexagenia cf. limbata | Canada, Ontario | 08ONMAY-0024 | DIMC123-09 | GU013604 |
| Caenidae | Caenis youngi | Canada, Ontario | 08ONMAY-0041 | DIMC124-09 | GU013576 |
| Leptophlebiidae | Leptophlebia cupida | Canada, Ontario | 08ONMAY-0064 | DIMC125-09 | GU013616 |
| Siphlonuridae | Siphlonurus alternatus | Canada, Ontario | 08ONMAY-0071 | DIMC126-09 | GU013660 |
| Ephemerellidae | Ephemerella aurivillii | Canada, Ontario | 08ONMAY-0072 | DIMC127-09 | GU013597 |
| Pseudococcidae | Planococcus citri | South Korea, Gyeonggido | mb-30 | DIMC130-09 |  |
| Pseudococcidae | Pseudococcus comstocki | South Korea, Jellanamdo | mb-02 | DIMC131-09 | GU013653 |
| Pseudococcidae | Planococcus kraunhiae | Japan | mb-20 | DIMC132-09 | GU013650 |
| Pseudococcidae | Phenacoccus aceris | South Korea, Jellanamdo | mb-34 | DIMC133-09 | GU013647 |
| Pseudococcidae | Crisicoccus matsumotoi | Japan | mb-24 | DIMC134-09 |  |
| Pseudococcidae | Dysmicoccus wistariae | South Korea, Gyeongsangnamdo | mb-41 | DIMC135-09 |  |
| Isotomidae* | Isotomiella | Canada, Manitoba | CHU06-COL-0503 | DIMC136-09 |  |
| Entomobryidae* | Entomobrya | Canada, Manitoba | CHU06-COL-0543 | DIMC137-09 |  |
| Isotomidae* | Isotomiella | Canada, Manitoba | CHU06-COL-0551 | DIMC138-09 | GU013613 |
| Onychiuridae* | Oligaphorura | Canada, Manitoba | CHU06-COL-0494 | DIMC139-09 | GU013639 |
| Isotomidae* | Isotomiella | Canada, Manitoba | CHU06-COL-0502 | DIMC140-09 | GU013612 |
| Onychiuridae* | Oligaphorura | Canada, Manitoba | CHU06-COL-0549 | DIMC141-09 | GU013638 |
| Onychiuridae* | Oligaphorura | Canada, Manitoba | CHU06-COL-0557 | DIMC142-09 | GU013637 |
| Isotomidae* | Folsomina | Gabon | 00623C01_GAB003 | DIMC143-09 | GU013611 |

Family names marked with a * are members of the Entognatha, while the remainder are members of the Insecta.
